# Quality of Life of Medical Students in Angola: A Cross-Sectional Study at Agostinho Neto University Using the World Health Organization Quality of Life-BREF (WHOQOL-BREF) Instrument

**DOI:** 10.7759/cureus.85613

**Published:** 2025-06-09

**Authors:** Cláudio Mbala, Carlos Tuti, Mário Fresta

**Affiliations:** 1 Cardiology, Multiperfil Clinic, Luanda, AGO; 2 Center for Advanced Studies in Medical Education and Training (CEDUMED), Agostinho Neto University, Luanda, AGO

**Keywords:** angola, medical education, medical students, quality of life, southern africa, whoqol-bref

## Abstract

Background

Medical training is globally recognized as a period marked by significant psychological and lifestyle challenges that may adversely impact students' quality of life (QoL). This issue is particularly critical in low-resource settings, such as Angola, where contextual stressors may further exacerbate these challenges and where no data on the QoL of medical students were previously available.

Aim

To assess the QoL and its determinants among medical students at Agostinho Neto University in Luanda, Angola.

Methods

A cross-sectional study was conducted using a convenience sample of 289 undergraduate medical students at the Faculty of Medicine of Agostinho Neto University (FMUAN) in Luanda, Angola. A cross-culturally adapted version of the World Health Organization Quality of Life-BREF (WHOQOL-BREF) questionnaire was used to assess QoL across four domains: physical, psychological, social relationships, and environment. Data were collected through self-completion of the questionnaire at the university facilities. Statistical analyses included descriptive statistics, t-test, ANOVA, chi-square tests, Pearson correlation, and multivariate regression, the latter of which was applied to identify factors associated with QoL domains.

Results

The sample consisted of 59.2% females, with a mean age of 26.09±6.99 years. Female students were more prevalent in younger age categories (p=0.001), and most students (84.8%) did not report chronic illnesses. The WHOQOL-BREF instrument demonstrated very good internal consistency (Cronbach’s alpha=0.840). Overall, QoL domain scores were low, with the environmental domain showing the lowest mean (45.19±15.70), followed by the physical domain (53.85±15.55). Conversely, the psychological domain had the highest mean score (70.30±11.52). Male students scored significantly lower in the social relationships (62.89 vs. 67.86) and environmental (40.81 vs. 47.14) domains, while female students scored lower in the psychological domain (68.40 vs. 71.63). QoL scores varied across academic years, with third-year students consistently reporting higher scores across most domains, whereas second- and sixth-year students reported the lowest scores. The presence of chronic illnesses was associated with lower QoL in the physical (p=0.016), psychological (p=0.015), and environmental (p=0.007) domains. Most students rated their overall QoL as “neither good nor bad” (51.9%), and about half were satisfied with their health (50.5%). Regression analysis showed that being in the third year and having better perceived overall QoL positively predicted QoL in the physical (β=14.54 and β=10.60, respectively), psychological (β=5.26 and β=5.64, respectively), and social domains (β=6.03 and β=16.27, respectively). Conversely, being a sixth-year student (β=−5.86) and being female (β=−3.72) negatively predicted physical and psychological QoL, respectively.

Conclusions

Our findings highlight generally low QoL scores among medical students in Angola, especially in the environmental and physical domains and notably during the initial and final academic years. Being in the third year and having a positive self-assessment of overall QoL emerged as protective factors, while female gender and being a sixth-year student were associated with poorer QoL. These insights underscore the necessity of targeted interventions to address the determinants of QoL and support student well-being in resource-constrained medical education settings such as Angola.

## Introduction

The concept of quality of life (QoL) is timeless, as the pursuit of a meaningful and healthy existence has been a central human aspiration since antiquity [1]. The World Health Organization (WHO) defines QoL as “an individual’s perception of their position in life in the context of the culture and value systems in which they live, and in relation to their goals, expectations, standards, and concerns” [2,3]. This definition highlights three key characteristics of QoL: subjectivity, multidimensionality, and bipolarity, encompassing both positive and negative aspects. In other words, QoL is shaped by the subjective evaluation of objective and subjective conditions; it spans multiple physical, psychological, and social domains and reflects desirable and undesirable elements [2].

The transition to higher education is widely considered a critical developmental phase, often marked by challenges, crises, and stressors [4]. This phase can lead to a temporary loss of control over the academic environment [4,5]. University students may experience significant changes in their mental health and lifestyle, such as increased consumption of alcohol, illicit drugs, and tobacco, poor dietary habits, lack of physical activity, and sleep disturbances - all of which can adversely affect their QoL and contribute to the development of chronic lifestyle-related diseases over time [6].

Medical school, in particular, has been described by some authors as a setting of “psychological toxicity” [1], prompting widespread research into the QoL of medical students across the globe [7-12]. Compared to their peers in other fields, medical students are exposed to additional stressors such as longer and more intensive training, heavier workloads, and the emotional burden of dealing with patient care, suffering, and death [4,5,13]. In the African context, students face further challenges, including inadequate infrastructure, limited social and financial support, and demanding academic environments, all of which may hinder their ability to cope with stress management and succeed academically [14]. A systematic review and meta-analysis of 31 studies estimated the prevalence of depression among African medical students at 38.8%, with lower rates reported among females and second-year students [15].

Furthermore, certain periods of medical training appear to be particularly conducive to psychological burden. The transition through the preclinical, clinical, and clerkship stages may influence students’ coping abilities, with the shift from basic science to clinical training often linked to increased demands [13]. Some studies have identified the early years of medical school as the most problematic [4,16,17], whereas others report higher stress levels during the clinical years, especially in transitional periods [18]. In the final years, students often face stress related to career uncertainty, the transition to professional practice, and escalating responsibilities [19].

Despite the expanding literature on QoL in the general population, there remains a notable gap in research focused on the QoL of medical students, particularly in Africa [5]. Given the specific and often intense challenges faced by this group, which can compromise the physical and mental health of future physicians, investigating their QoL is critical in the context of contributing to proposals and strategies for the improvement of medical education and training in Southern Africa - particularly in Angola. Therefore, this study aimed to assess the QoL and its determinants among medical students of the Agostinho Neto University in Luanda, Angola.

## Materials and methods

Study design and settings

A cross-sectional study was conducted from July to August 2010 among medical students at the Faculty of Medicine of Agostinho Neto University (FMUAN), the first and main public medical school in Angola. At that time, there were only two medical schools in the country.

The study was performed in accordance with the Strengthening the Reporting of Observational Studies in Epidemiology (STROBE) guidelines.

Study participants

The study population consisted of all 729 undergraduate medical students enrolled in FMUAN during the 2010 academic year. A convenience sample was selected, consisting of students from the first, second, third, and sixth graduation years, who voluntarily agreed to participate. It was decided to include these graduation years due to their specific characteristics, such as years that mark the beginning or transition of the different cycles that constitute the course of medicine in this university. Thus, these years represent the beginning, middle, and end of the course. Moreover, students from the fourth and fifth years were not included due to logistical constraints at the time.

Study participants were recruited according to the following inclusion criteria: students with active enrollment in the 2010 academic year, students in their first, second, third, or sixth academic year, and students who voluntarily agreed to participate in the study by signing the informed consent form. The exclusion criterion was questionnaires with more than 20% of unanswered questions, as recommended by the World Health Organization Quality of Life (WHOQOL) Group [3].

Sampling and sample size calculation

As previously described, a convenience sample was used in this study. The sample size was calculated based on a population of 729 students, with a 95% confidence interval, an expected frequency of 50%, and a 5% margin of error, resulting in an estimated 252 individuals. To account for a potential loss of 10-15% of individuals, the sample size was increased to 289 individuals, which corresponded to approximately 72% of the students enrolled in the academic years included in the study. An online calculator was used for this purpose [20].

Study tool

The instrument used to assess students' QoL was the World Health Organization (WHO) generic questionnaire on QoL, the WHOQOL-BREF, which is the abbreviated version of the WHOQOL-100, both developed by the WHO Quality of Life Group (WHOQOL Group).

The short version was preferred due to its brevity and psychometric adequacy [21].

The European Portuguese version of the WHOQOL-BREF was developed by the Portuguese WHOQOL Center (Portugal) in 2006 and has demonstrated good psychometric properties [21]. In the present study, we used an adapted version of the European Portuguese questionnaire due to its closer linguistic resemblance to the Portuguese spoken in Angola. Permission to use the instrument was granted by both the WHO [3] and the Portuguese WHOQOL Group [21].

The WHOQOL-BREF consists of 26 items, including two general questions, one on overall QoL and the other on general health satisfaction, and 24 additional questions related to four domains: physical, psychological, social relationships, and environment. The questionnaire consists of multiple-choice closed-ended questions, designed for easy administration, and considers the respondents' experiences over the past two weeks. The questions are structured using four types of five-point Likert scales: intensity (Not at all - Extremely), capacity (Not at all - completely), frequency (Never - Always), and evaluation (Very satisfied - Very dissatisfied; Very bad - Very good) [2,3]. To enhance interpretability, the response extremes were combined (e.g., “Dissatisfied” and “Very dissatisfied”; “Good” and “Very good”).

The physical domain (seven items) focuses on the following facets: pain and discomfort, energy and fatigue, sleep and rest, mobility, activities of daily living, dependence on medication or treatments, and work capacity. The psychological domain (six items) includes the following facets: positive feelings, learning, memory, concentration, self-esteem, body image and appearance, negative feelings, spirituality, religion, and personal beliefs, while the social relationships domain (three items) encompasses personal relationships, social support, and sexual activity. Finally, the environment domain (eight items) is related to physical safety, home environment, financial resources, healthcare and social support, opportunities to acquire new information, opportunities for recreation and leisure, physical environment, and transportation [2].

Results are calculated using specific equations and then converted into scores ranging from 4-20 and/or 0-100 to ensure comparability with the longer version, the WHOQOL-100, with lower scores indicating poorer QoL and higher scores indicating better QoL. Although there seems to be no consensus in the literature regarding cutoff points, some authors consider a score of 60 or higher to indicate a good QoL [11,22].

Cross-cultural adaptation and pilot study

The adaptation process of the questionnaire followed international guidelines [23] to ensure linguistic and cultural equivalence. The process included forward and backward translation (adaptation), expert review, and a pilot study. At the end of the process, it was unanimously decided to retain all 26 questions in a form like the original questionnaire, as they were deemed fully comprehensible to the target population - medical students. Only minor modifications were made, such as adjustments to the cover page and the exclusion of certain questions related to respondents' personal data, which were considered unnecessary for the present study (e.g., education level, occupation, address, and marital status). The academic year was added as a relevant variable.

With the adapted version finalized, a pilot study was then conducted by administering the questionnaire to 35 students who were not included in the main study to assess comprehension, interpretation, and acceptability. All distributed questionnaires were completed and returned (100% response rate), and 86% were fully filled out. All participants reported the questionnaire as clear and comprehensible. No suggestions for improvements were made. The adapted questionnaire thus demonstrated good validity, as well as good internal consistency and reliability, with an overall Cronbach's alpha of 0.780.

Data collection

The survey took place during the last weeks of the first academic semester. The questionnaire was self-administered by students who voluntarily agreed to participate in the study, in their classrooms between classes. Beforehand, the first author provided a detailed explanation of the study’s objectives and comprehensive instructions for completion. The author was present solely to offer clarifications if needed, ensuring no influence on the students’ responses.

Ethical considerations

The study was approved by the Institutional Review Board of the Faculty of Medicine of Agostinho Neto University. Prior to data collection, participants were thoroughly informed about the study's objective, the questionnaire administration procedures, the voluntary nature of participation and the right to withdraw at any time, and the assurance of data anonymity. All individuals who agreed to participate provided written informed consent.

Data analysis

Data were manually collected and organized in a Microsoft Excel (Microsoft® Corp., Redmond, WA) spreadsheet. Subsequently, statistical analysis was performed using Statistical Product and Service Solutions (SPSS, version 28.0; IBM SPSS Statistics for Windows, Armonk, NY). For descriptive statistics, categorical variables were described as frequencies and percentages, while continuous variables were presented as means and standard deviations (SD). The Kolmogorov-Smirnov test was used to assess data normality. Student T-test and ANOVA were used to detect differences between domain scores among subgroups. Pearson’s correlation test was used to assess the correlation between the different WHOQOL-BREF domains. The chi-square test was used to determine differences between categorical variables. A multivariate linear regression model was employed to identify factors independently associated with QoL domain scores. Statistical significance was set at p < 0.05.

## Results

The sample’s demographic characteristics

A total of 289 students participated in the study, of whom 171 (59.2%) were female. The mean age of the participants was 26.04 years (SD=6.99), with male students being significantly older than their female counterparts (27.23±6.67 vs. 25.22±7.10 years; p=0.016). There was a statistically significant association between age group and gender (p=0.001). Female students were more frequently under 20 years old (21.6%) compared to males (5.1%), whereas a higher proportion of male students were aged 30-39 years (22.9% vs. 14.6%). Regarding academic year distribution, no statistically significant difference was found between male and female students (p=0.796), suggesting a balanced gender distribution across the selected years (first, second, third, and sixth). Similarly, the presence of chronic diseases did not differ significantly by gender (p=0.187), although female students reported a slightly higher prevalence (17.5%) compared to males (11.9%). These sociodemographic characteristics of the students are summarized in Table 1.

**Table 1:** Sociodemographic, academic, and health status of the sample (n=289) SD-Standard deviation; *t-test; **Chi-square test

Variables	Gender		p-value
	Male n=118 (40.8%)	Female n=171 (59.2%)	
Age - mean (SD)	27.23 (6.67)	25.22 (7.10)	0.016*
Age group, n (%)			
< 20 years	6 (5.1%)	37 (21.6%)	0.001**
20–29 years	76 (64.4%)	98 (57.3%)	
30–39 years	27 (22.9%)	25 (14.6%)	
≥ 40 years	9 (7.6%)	11 (6.4%)	
Academic year, n (%)			
1st Year	35 (29.7%)	51 (29.8%)	0.796**
2nd Year	31 (26.3%)	37 (21.6%)	
3rd Year	30 (25.4%)	50 (29.2%)	
6th Year	22 (18.6%)	33 (19.3%)	
Presence of chronic disease, n (%)			
Yes	14 (11.9%)	30 (17.5%)	0.187**
No	104 (88.1%)	141 (82.5%)	

Instrument’s reliability

The instrument demonstrated very good internal consistency in this sample, with Cronbach’s alpha of 0.840 for all 26 items. Cronbach’s alpha values for the physical, psychological, social relationships, and environmental domains were 0.687, 0.597, 0.477, and 0.785, respectively.

Overall scores of WHOQOL-BREF among medical students

**Figure 1:**
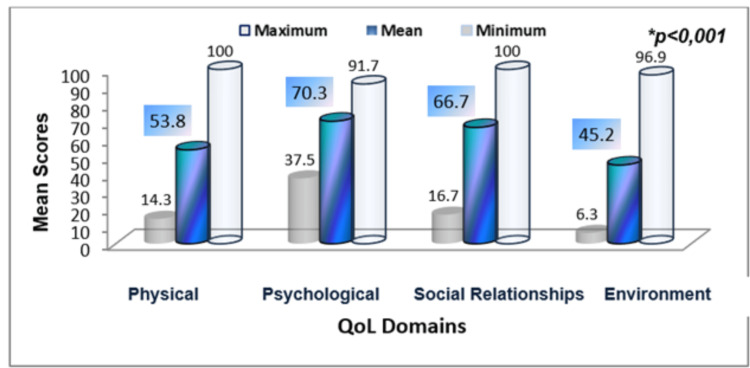
Overall mean scores of the students in the quality of life domains * t-test

Overall, the WHOQOL-BREF quality of life domain scores for FMUAN students demonstrated low scores in nearly all domains (see Figure 1).

The lowest mean score was observed in the environment domain (45.19±15.70), which also presented the widest score range (6.25-96.88). The physical domain was the second lowest rated, with a mean score of 53.85±15.55. On the other hand, the highest mean score was observed in the psychological domain, with 70.30±11.52. Comparative analysis across the four domains showed that the differences in mean scores were statistically significant (p<0.001).

Assessment of QoL domains according to sociodemographic, academic, and health-related factors

Male students presented lower mean scores (62.89 vs. 67.86) in the social relationships (p=0.021) and, particularly, the environmental (40.81 vs. 47.14) domains (p<.001), while female students had significantly lower scores (52.99 vs. 55.77) in the psychological domain (p=0.039). These findings suggest the impact of gender on the perception of QoL in multiple aspects.

While no statistically significant differences were observed in QoL domain scores across different age groups, some notable trends emerged. The environmental domain consistently yielded the lowest numerical means overall, with this trend being particularly pronounced in the older age groups (41.79±14.14). Conversely, the psychological domain showed the highest numerical mean scores, demonstrating a tendency towards better results among older students (70.32±13.08). However, younger students (< 20 years) reported slightly better scores in the social relationships (68.49±15.42) and environmental (49.76±14.07) domains.

The assessment of QoL domains varied considerably across academic years. In general, third-year students reported the highest average scores across almost all domains, whereas second- and sixth-year students had the lowest. In the physical domain, third-year students had significantly (p<0.001) higher mean scores (66.75±14.18) compared to students in the first-year (50.24±12.83), second-year (55.25±15.82), and, especially, sixth-year (46.80±12.66) students. In the psychological domain, third-year students also had significantly (p=0.003) better QoL evaluation (75.14±10.96) than first-year (68.50±10.23), second-year (67.22 ± 16.46), and sixth-year students (68.69±12.36). In the social relationships domain, there was a marginal difference (p=0.046) between third-year students’ mean score (70.10±18.30) and their peers in the first (63.66±17.93), second (62.12±16.72), and sixth (67.41±17.10) academic years. In the environmental domain, all groups presented very low mean scores, without significant differences, although there was a numerical trend for third-year students to have higher mean scores and second- and sixth-year students the lowest.

The assessment of QoL domains revealed considerable variability across academic years. Overall, third-year students reported the highest average scores across almost all domains, while second- and sixth-year students consistently exhibited the lowest. Specifically, in the physical domain, third-year students had significantly higher mean scores (66.75±14.18) compared to first-year (50.24±12.83), second-year (55.25±15.82), and particularly sixth-year (46.80±12.66) students (p<0.001). Similarly, in the psychological domain, third-year students had a significantly higher mean score (75.14±10.96) than their peers in the first (68.50±10.23), second (67.22±16.46), and sixth (68.69±12.36) years (p=0.003). For the social relationships domain, a marginal statistically significant difference (p=0.046) was observed, with third-year students' mean score (70.10±18.30) being higher than that of first (63.66±17.93), second (62.12±16.72), and sixth (67.41±17.10) academic years. In the environmental domain, there was a numerical trend for third-year students to have higher scores and second- and sixth-year students to have the lowest, although these differences did not reach statistical significance. These findings may suggest poorer QoL at the start of medical school, some adaptation and stabilization in the middle years, and possible wear and tear toward the end of the course.

Students with chronic diseases reported significantly lower scores in the physical (p=0.016), psychological (p=0.015), and environmental (p=0.007) domains, underscoring the direct negative impact of these conditions on multiple dimensions of perceived QoL.

A comprehensive summary of comparisons across sociodemographic, academic, and health-related variables is provided in Table 2.

**Table 2:** Quality of life domains and sociodemographic, academic, and health-related factors among students P-value significant < 0.05; t-test or ANOVA was used.

Variables	Physical Domain		Psychological Domain		Social Relationships Domain		Environmental Domain	
	Mean (SD)	p-value	Mean (SD)	p-value	Mean (SD)	p-value	Mean (SD)	p-value
Gender								
Male	55.77 (16.22)	0.141	71.63 (11.99)	0.039	62.89 (17.07)	0.021	40.81 (14.60)	<0.001
Female	52.99 (15.07)		68.40 (12.94)		67.86 (17.93)		47.14 (15.25)	
Age Group								
<20 years	52.40 (15.07)	0.514	67.26 (10.10)	0.600	68.49 (15.42)	0.584	49.76 (14.07)	0.094
20-29 years	55.16 (16.21)		70.13 (13.29)		65.77 (17.60)		44.10 (15.23)	
30-39 years	51.86 (15.25)		70.32 (13.08)		63.46 (19.11)		41.79 (14.14)	
≥ 40 years	54.46 (15.07)		69.90 (10.84)		67.08 (19.77)		44.07 (19.14)	
Academic year								
1^st^ Year	50.24 (12.83)	<0.001	68.50 (10.23)	0.003	63.66 (17.93)	0.045	44.14 (14.20)	0.134
2^nd^ Year	55.25 (15.82)		67.22 (16.46)		62.12 (16.72)		41.78 (13.17)	
3^rd^ Year	66.75 (14.18)		75.14 (10.96)		70.10 (18.30)		48.25 (14.89)	
6^th^ Year	46.80 (12.66)		68.69 (12.36)		67.41 (17.10)		43.87 (17.75)	
Chronic disease								
Yes	48.94 (14.82)	0.016	64.48 (15.01)	0.015	62.31 (16.61)	0.075	39.21 (16.35)	0.007
No	55.09 (15.56)		70.66 (11.94)		66.49 (17.87)		45.48 (14.91)	

Overall quality of life assessment

**Figure 2:**
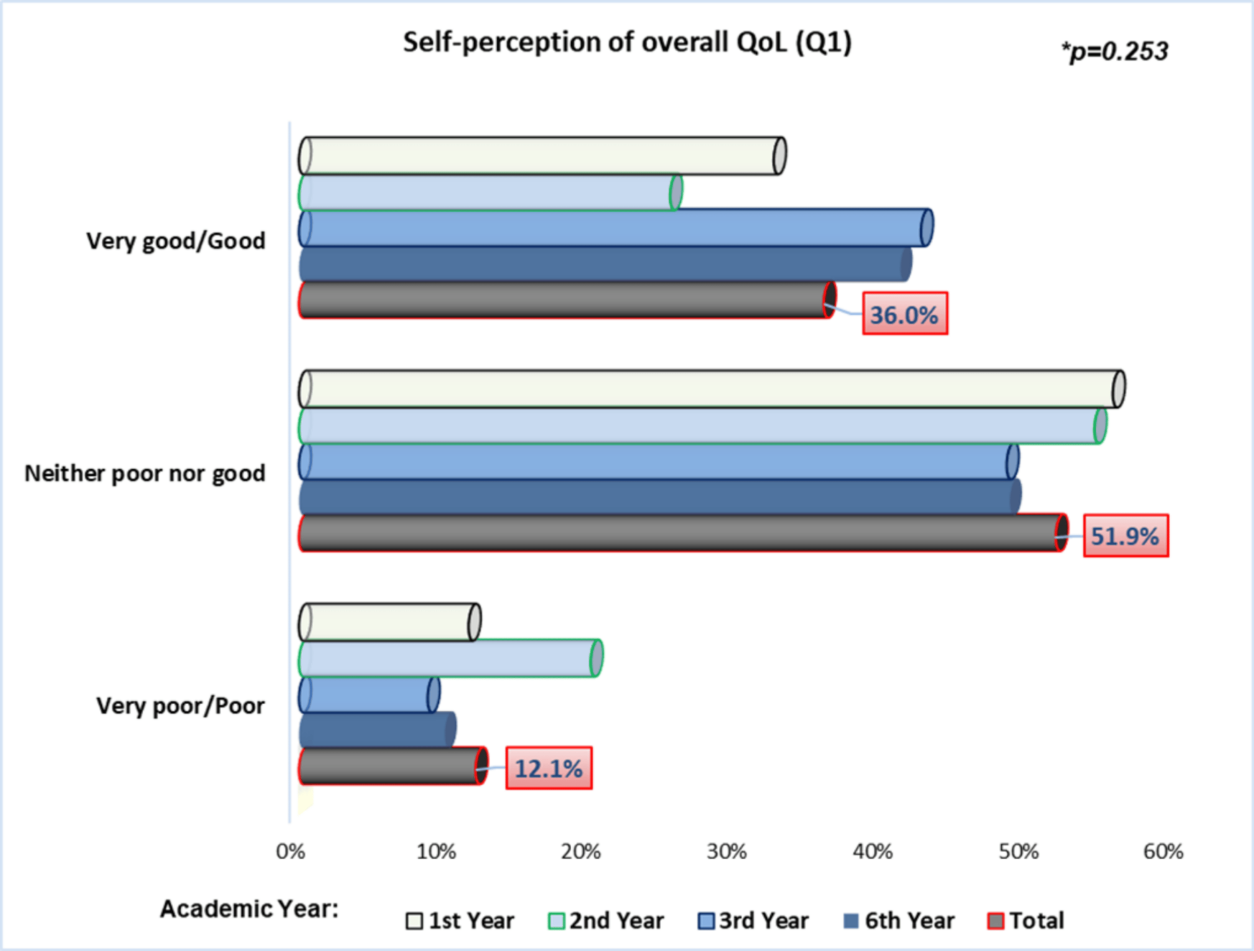
Students’ perception of their overall quality of life *Chi-square test

Regarding the first general question of the WHOQOL-BREF “How would you rate your quality of life?”, the majority of students (51.9%) self-reported their QoL as “Neither good nor poor”; it is also noteworthy that 12.1% considered their QoL to be “Very poor” or “Poor”, with first- and second-year students predominating in this group. Conversely, approximately 36.0% of the students reported that their QoL was “Good” or “Very good”. Third-year students tended to report a better self-perception of their QoL, although without a statistically significant difference (p=0.253), using the chi-square test (Figure 2). The mean score for this item was 3.23 (±0.75).

**Figure 3:**
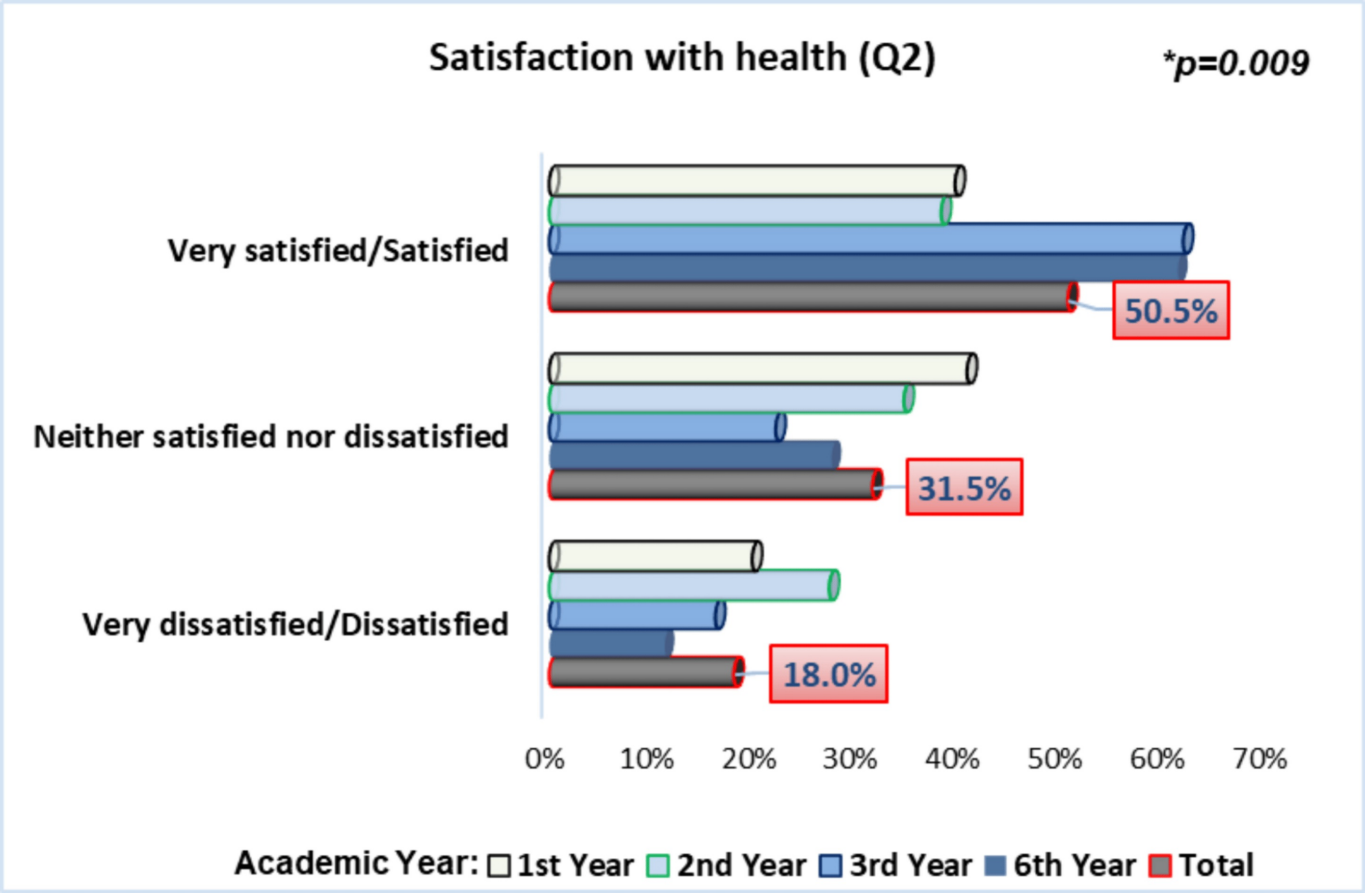
Students’ satisfaction with their health *Chi-square test

Proceeding to the second general question of the WHOQOL-BREF “How satisfied are you with your health?”, slightly over half of the students (50.5%) reported being "Satisfied" or "Very Satisfied" with their health, particularly those in the third (61.7%) and sixth years (61.2%), who showed significantly greater satisfaction with their own health compared to their peers (p=0.009), using the chi-square test (Figure 3). The mean score for this question was 3.37 (±0.89).

Bivariate correlation between QoL domains

The Pearson's correlation analysis showed that all WHOQOL-BREF domains were positively and significantly correlated with each other, as well as with the general questions (p < 0.001), as shown in Table 3. The strongest correlations were observed between the psychological and physical health domains, followed by the correlation between the environmental health domain and the general Question (Q1) on the overall self-assessment of QoL.

**Table 3:** Bivariate correlation between students' quality of life domains The results are presented as: Pearson's correlation coefficient (p-value). Significance at the 0.01 level.

Variable	Physical Domain	Psychological Domain	Social Domain	Environmental Domain
Q1 (Overall QoL)	0.396 (<0.001)	0.228 (<0.001)	0.328 (<0.001)	0.480 (<0.001)
Q2 (Health Satisfaction)	0.295 (<0.001)	0.325 (<0.001)	0.226 (<0.001)	0.291 (<0.001)
Physical Domain	----	0.523 (<0.001)	0.360 (<0.001)	0.394 (<0.001)
Psychological Domain	0.523 (<0.001)	----	0.318 (<0.001)	0.328 (<0.001)
Social Domain	0.360 (<0.001)	0.318 (<0.001)	----	0.396 (<0.001)
Environmental Domain	0.394 (<0.001)	0.328 (<0.001)	0.396 (<0.001)	----

Factors associated with QoL among medical students

A multivariate analysis using multiple linear regression was performed to identify factors associated with QoL domains (Table 4).

**Table 4:** Multivariate analysis of factors associated with the different domains of students' QoL Ref. - Reference category. QoL - Quality of life The results are presented as regression coefficient (95% confidence interval); *p<0.05; **p<0.01

Variables	Physical Domain β (95% CI)	Psychological Domain β (95% CI)	Social Domain β (95% CI)	Environmental Domain β (95% CI)
Student's Age				
Ref. <25 years	---	---	---	---
≥ 25 years	1.89 (-1.85; 5.63)	0.18 (-3.30; 3.67)	-2.78 (-7.62; 2.07)	-0.16 (-4.07; 3.74)
Gender				
Ref. Male	---	---	---	---
Female	-2.95 (-6.19; 0.28)	-3.72 (-6.69; -0.76) *	2.62 (-1.50; 6.74)	3.75 (0.39; 7.10) *
Presence of Disease				
Ref. Yes	---	---	---	---
No	1.91 (-2.63; 6.45)	1.58 (-2.67; 5.83)	1.31 (-4.57; 7.18)	1.76 (-3.01; 6.54)
Academic Year				
Ref. 1st Year	---	---	---	---
2nd Year	5.94 (1.56; 10.31) *	-0.36 (-4.39; 3.67)	0.70 (-5.53; 5.67)	-0.70 (-5.23; 3.82)
3rd Year	14.54 (10.25;18.82) **	5.26 (1.21; 9.31) *	6.03 (0.47; 11.58) *	2.53 (-1.96; 7.03)
6th Year	-5.86 (-10.64; -1.08) *	-1.16 (-5.53; 3.21)	4.58 (-1.58; 10.73)	-1.86 (-6.79; 3.08)
Overall QoL Assessment				
Ref. Very poor/poor	---	---	---	---
Neither good nor poor	7.70 (2.63; 12.77) *	3.47 (-1.09; 8.03)	12.99 (6.56; 19.43) **	8.88 (3.67; 14.08) **
Good/Very good	10.60 (4.96; 16.24) **	5.64 (0.57; 10.70) *	16.27 (9.06; 23.48) **	18.28 (12.51; 24.05) **
Satisfaction With Health				
Ref. Very Dissat./Dissatisfied	---	---	---	---
Neither Sat./Nor Dissatisfied	3.02 (-1.66; 7.69)	3.34 (-0.97; 7.66)	-1.96 (-8.13; 4.21)	2.14 (-2.78; 7.08)
Satisfied/Very Satisfied	5.62 (0.78; 10.46) *	6.73 (2.27; 11.19) *	1.35 (-5.11; 7.80)	4.43 (-0.63; 9.48)

Regarding the physical domain, being a second-year student (β=5.94; 95% CI: 1.56-10.31) and particularly a third-year student (β=14.54; 95% CI: 10.25-18.82) was significantly associated with higher scores in this domain compared to first-year students (p<0.01). Conversely, being a sixth-year student (β=-5.86; 95% CI: -10.64 to -1.08) was associated with lower scores (p<0.05). Thus, both first- and sixth-year students demonstrated poorer physical well-being. Students who reported a better overall QoL (β=10.60; 95% CI: 4.96-16.24; p<0.01) and greater satisfaction with their health (β=5.62; 95% CI: 0.78-10.46; p<0.05) were also associated with higher scores in this domain.

Being a female student was associated with lower scores in the psychological health domain (β=-3.72; 95% CI: -6.69 to -0.76; p<0.05). Conversely, being a third-year student (β=5.26; 95% CI: 1.21-9.31; p<0.05), reporting a better overall QoL (β=5.64; 95% CI: 4.96-16.24; p<0.05), and being satisfied with one's health (β=6.73; 95% CI: 2.27-11.19; p<0.05) were associated with better psychological health.

Better QoL in the social relationships domain was associated with being a third-year student (β=6.03; 95% CI: 0.47-11.58; p<0.05) and with a better self-assessment of overall QoL (β=16.27; 95% CI: 9.06-23.48; p<0.01).

Regarding the environmental health domain, the factors associated with better QoL were being a female student (β=3.75; 95% CI: 0.39-7.10; p<0.05) and reporting a better self-assessment of overall QoL (β=18.28; 95% CI: 12.51-24.05; p<0.01).

## Discussion

This study represents the first assessment of the QoL among medical students in Angola, a country located in sub-Saharan Africa, specifically in Southern Africa.

The 289 evaluated students were predominantly female, a finding consistent with other similar studies conducted in Africa [7,8], Europe [24], South America [19], and Asia [11]. This trend reinforces the growing predominance of female students in medical schools and, consequently, in the medical profession [8].

The mean age was 26.04 years (±6.99), with a predominance in the 20-24 age group (36%), although the majority of students (49.1%) were aged above 24 years. While similar age distributions were observed in some studies [8], most previous research reported younger medical students [10,24].

Most students self-reported being healthy, a pattern also observed in other studies [7,9,10,25].

In the present sample, the WHOQOL-BREF demonstrated very good internal consistency (Cronbach's alpha of 0.840), similar to several other studies involving medical students [7,10,12]. However, the social relationships domain exhibited a lower Cronbach's alpha (0.477), a pattern also observed in another research [8]. This may be attributed to its relatively limited number of facets (only three items, compared to five to eight items in other domains) and its broad conceptual variability (encompassing aspects such as social support and sexual activity), which can lead to lower inter-item correlations and, consequently, reduced internal consistency. These findings reflect the inherent challenges of measuring a multifaceted concept with a limited number of items.

Regarding the overall assessment of the WHOQOL-BREF domain scores, students demonstrated predominantly low mean scores across almost all domains, with the environmental domain being the most negative, followed by the physical domain, while the psychological domain had the highest scores. Some studies have also reported similar mean scores [24], as well as a lower evaluation in the environmental domain, as seen in studies conducted in Brazil [1], South Korea [12], and especially in Egypt [7] and Sudan among medical and health sciences students from over 80 African countries [8].

The environmental health domain includes a wide range of facets encompassing multiple aspects of daily life, which, in today's world, marked by consumerism in capitalist societies, and poverty and other socioeconomic challenges in developing countries, may contribute to more negative evaluations. Students' perceptions of lower environmental QoL are therefore linked to a combination of factors, such as physical insecurity, financial difficulties, poor or limited access to healthcare services, lack of social support, insufficient recreational activities, and inadequate transportation systems [24]. Angola continues to grapple with deep-rooted socioeconomic inequalities, significantly affecting various aspects of public welfare and healthcare access, resulting in persistently high infant mortality rates [26]. In light of this challenging social context, it is thus understandable that students reported poor environmental QoL. Human, infrastructural, and socioeconomic barriers remain major obstacles for health sciences students in sub-Saharan Africa [14], as well as in other regions such as Central Europe [25]. Interestingly and paradoxically, a few studies reported positive evaluations of the environmental domain among medical students [9-11].

Although the psychological domain was the highest-rated domain among students in the present study, this finding contrasts with most other research [10,22], where the literature is nearly unanimous in highlighting the presence of potentially stressful factors during medical training. Medical students are widely recognized to be at greater risk of developing psychological disorders [9], many of which are linked to frequent exposure to critically ill patients and death, and highly demanding evaluation methods, among other stressors [6,15,18].

Male students had the lowest scores in the social relationships and, more markedly, the environmental domains. This result, in line with the broader discussion previously presented regarding this domain, may be explained by the fact that social demands continue to weigh more heavily on men, who represent the majority of working students with family responsibilities [27]. However, contrasting results have been reported among medical students in Saudi Arabia [11]. Female students, in turn, demonstrated poorer scores in the psychological and physical domains, as observed in many previous studies [27,28]. Other studies reported even broader impacts, showing that female students had lower QoL scores across all domains [1,12]. Overall, our findings are in strong agreement with much of the literature, which is nearly unanimous in reporting that female students exhibit higher levels of stress compared to their male counterparts [19]. Conversely, an important systematic review on the prevalence of depression among medical students in Africa showed that being female was associated with a lower prevalence of depression [15]. Furthermore, some authors argued that there are no significant sex differences in the perception of stress or other psychological, physical, or QoL outcomes [1,7,10].

Concerning the distribution of QoL mean scores by age group, no statistically significant differences were observed, consistent with findings from other studies [9]. Nevertheless, in the psychological domain, there was a numerical trend toward higher scores among older students. Regardless of age, medical students represent a particularly vulnerable group to stress, burnout, depression, and anxiety [6,13,15]. However, older students appear to develop better academic stress coping strategies [8,12].

Regarding the assessment of QoL according to academic year, third-year students presented the best evaluation in all domains. In the physical domain, the lowest mean score was observed among sixth-year students, whereas in the psychological domain, it was among second- and first-year students. The social relationships domain had the worst evaluation among second-year students. In the environmental domain, all means were very low, with the sixth year numerically obtaining the lowest score, although without statistical significance. These results are similar to those found in several studies [4,9,18].

Lower QoL scores during the early years and in the final year of medical school are strongly supported by existing literature. Students in the early stages of training, many of whom relocate from different regions, often face the challenge of adapting to a completely new environment. This transition involves significant lifestyle changes, such as new dietary habits, altered daily schedules, and unfamiliar living conditions, many of which may differ substantially from their previous routines [16]. In line with this, a study conducted in Indonesia found that first-year medical students had higher rates of depression and anxiety [17]. Furthermore, being in the sixth year of medical training has been identified as a potential negative predictor of QoL [19]. It is important to note, however, that some studies involving African medical students did not observe significant differences in QoL across academic years [8], suggesting that medical students may experience relatively stable levels of stress and academic pressure throughout their training.

At the time of this study, FMUAN was the only public medical school in Angola, attracting a diverse student population from across the country. This was particularly relevant for first-year students, many of whom relocated to the capital city often without adequate family or social support, potentially increasing psychosocial stress and negatively impacting their QoL. In contrast, sixth-year students faced substantial clinical responsibilities, directly confronting the complex and resource-limited healthcare challenges of the country. This heightened pressure and accountability likely contribute to lower QoL scores in this group. Moreover, it is important to note that not a few of these students have already been enrolled in medical school for more than six years, due to academic failures or interruptions in classes. Additionally, many sixth-year students were older, often with significant family and social responsibilities and, in some cases, working while studying. Third-year students, however, have usually adapted socially and academically to their new environment and the demands of medical training. Moreover, the introduction of supervised clinical practice and the opportunity to engage with patients after two years of basic sciences can significantly enhance their motivation and satisfaction. This renewed sense of purpose and practical experience likely fosters higher QoL in third-year students compared to their peers. Taken together, these local contextual factors, alongside the broader socioeconomic and healthcare system challenges in Angola, offer important insights into the variability of QoL scores across academic years in our study. Addressing these specific stressors could inform targeted interventions to improve medical students’ well-being throughout their training.

Regarding the relationship between students' health status and their QoL, those with chronic illnesses reported lower scores across all WHOQOL-BREF domains, except for the social relationships domain, in which there was no significant difference. Similar findings have been reported among medical students in Egypt [7], Brazil [9], and Poland [16], reinforcing the literature that students with chronic diseases are more likely to experience a poorer QoL [29].

Concerning the self-assessment of overall QoL, the majority of students rated it as “Neither good nor bad”, while a small proportion perceived it as “Very poor” or “Poor”, mainly among first- and second-year students. In contrast, only approximately one-third classified their overall QoL as “Good” or “Very good”, particularly among third-year students. These results are lower than those reported in many other studies, conducted in Egypt [7], Portugal [24], and Spain [10].

As for general satisfaction with health, although about half of the students reported being “Satisfied” or “Very satisfied”, this proportion can be considered relatively modest. Additionally, approximately one-fifth reported being “Very dissatisfied” or “Dissatisfied” with their health, mainly among first- and second-year students. In contrast, students in the third and sixth years stood out for reporting more positive self-assessments of their health. It is possible that the more direct and frequent contact and engagement with patients and various pathologies, with greater responsibility, make sixth and third-year students more resilient, leading them to have a more positive perception of their own health. In other studies, the frequency of students satisfied with their health was higher [7,10,24].

The QoL domains showed a positive and statistically significant correlation with each other, as well as with the general items (Q1 and Q2). The strongest correlations were observed between the psychological and physical domains, followed by the correlation between the environmental domain and the general item (Q1) on self-assessment of overall QoL. Pearson's correlation coefficients ranged from 0.226 to 0.523. Similar results were found in other studies [7,10].

Through multiple linear regression, the predictors of higher QoL were being a third-year student (in almost all domains), having a positive self-perception of one’s overall QoL (in all domains), being satisfied with one's health (psychological domain), and being female (only in the environmental domain). Conversely, predictors of lower QoL included being a sixth and first-year student, being female (psychological domain), and having a negative self-perception of one's overall QoL. Similar findings have been reported in studies conducted in Spain and South Korea, where being female was also associated with poorer QoL in the psychological domain [10,12]. A positive self-assessment of overall QoL and health as predictors of better QoL across several domains has also been observed among medical students in Brazil [22], Poland [25], and Portuguese higher education students [24]. In contrast, other studies found no significant association between QoL and variables such as gender, age, or academic year among medical students [30].

Limitations

This study has several limitations that should be acknowledged. First, the data were collected in 2010, and although they provide valuable baseline insights, they may not fully reflect the current context of medical education in Angola, which has likely undergone substantial changes over the past decade. Second, the exclusion of fourth- and fifth-year medical students - due, in part, to operational constraints at the time of data collection - may have limited a more comprehensive representation of all academic years. Although third-year students may partially reflect this stage of training, their experiences might not fully capture the perspectives of more senior students. Third, relevant variables potentially associated with QoL - such as sleep duration, physical activity, depressive symptoms, academic performance, and socioeconomic status - were not assessed and should be considered in future studies to provide a more holistic understanding. Fourth, the use of a non-probabilistic sampling approach may affect the external validity of the findings. Additionally, as this was a cross-sectional study, causal relationships between the investigated factors and QoL cannot be established.

Despite these limitations, this remains, to the best of our knowledge, the first and only study to assess the QoL of medical students in this local context. The findings offer important preliminary insights and may serve as a valuable basis for longitudinal, multicenter, and qualitative studies aimed at elucidating the determinants of students’ well-being from their own perspectives and informing targeted interventions to improve their QoL.

## Conclusions

The QoL among medical students in Angola is relatively low, particularly in the environmental and physical domains, and notably during the early and final academic years. Third-year students reported the highest QoL scores, possibly reflecting better adaptation and improved well-being over time - until a notable decline in the sixth year. Medical schools and related institutions should actively monitor students’ well-being and implement measures to reduce academic overload, especially in the first and final years, through a careful review and adjustment of the academic schedule. It is also essential to ensure access to psychological and social support services and to promote healthier study environments. Encouraging balanced time management and self-care practices may not only improve students’ QoL but also enhance their academic performance and, consequently, their long-term professional development in the future.
